# Atypical Persistent Pruritic Hyperpigmented Plaques in Adult‐Onset Still's Disease: A Case Report From Uganda

**DOI:** 10.1002/ccr3.72668

**Published:** 2026-05-10

**Authors:** Abdisalam Ahmed Sandeyl, Stephen Kizito Mirembe, Kusemererwa Byaruhanga, Farah Dubad Abdi, Abdisamad Guled Hersi, Louis K. Kamyuka, Venance Emmanuel Mswelo, Rawan Yousif Hassan

**Affiliations:** ^1^ Internal Medicine Department Kampala International University, Western Campus Ishaka‐Bushenyi Uganda; ^2^ Dermatology Department Kampala International University, Western Campus Ishaka‐Bushenyi Uganda

**Keywords:** adult‐onset Still's disease, atypical cutaneous manifestations, autoinflammatory disorders, persistent pruritic hyperpigmented plaques, Uganda, Yamaguchi criteria

## Abstract

We report a case of adult‐onset Still's disease (AOSD) in a 30‐year‐old Ugandan man presenting with migratory polyarthralgia, intermittent fever, lymphadenopathy, and atypical persistent pruritic hyperpigmented plaques. Laboratory evaluation showed neutrophilic leukocytosis, markedly elevated C‐reactive protein, and hyperferritinemia. Infectious and autoimmune causes were excluded. A diagnosis of moderate systemic AOSD was established based on the Yamaguchi criteria. Treatment with corticosteroids and nonsteroidal anti‐inflammatory drugs led to rapid clinical improvement and progressive regression of the cutaneous lesions on follow‐up. This case highlights the need for considering atypical persistent dermatologic manifestations of AOSD, especially in low‐resource settings.

## Key Clinical Message

Adult‐onset Still's disease may present with persistent atypical hyperpigmented skin lesions rather than the classic evanescent rash. Recognition of such atypical cutaneous manifestations in the context of systemic inflammation is essential for timely diagnosis and management, particularly in resource‐limited settings.

## Graphical abstract

Atypical Persistent hyperpigmented cutaneous lesions associated with adult‐onset Still's disease.

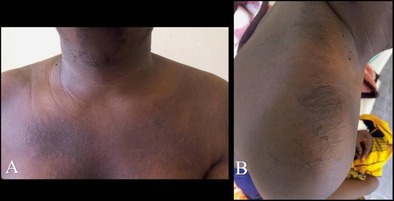

**Abbreviations**
AOSD: adult‐onset Still's disease
CRP: C‐reactive protein
IL: interleukin

## 1 Introduction

Adult‐onset Still's disease (AOSD) is a rare systemic autoinflammatory disorder characterized by quotidian fever, polyarthritis, and distinctive dermatologic manifestations [1]. The classic rash is transient, salmon‐pink, and evanescent, often coinciding with fever spikes. In contrast, atypical cutaneous manifestations, including persistent pruritic hyperpigmented or violaceous plaques, have been increasingly recognized as part of the disease spectrum. Such persistent lesions may mimic eczema, drug eruptions, urticarial vasculitis, or other chronic dermatoses, leading to significant diagnostic delays, particularly in settings with limited access to dermatopathology [2]. Reports of persistent atypical cutaneous manifestations of AOSD from sub‐Saharan Africa remain scarce [3]. We report a case of AOSD presenting with persistent pruritic hyperpigmented plaques in a young Ugandan man, emphasizing diagnostic challenges and clinical recognition in a resource‐limited setting.

## 2 Case Presentation

### 2.1 Case History and Examination

A 30‐year‐old man with no prior chronic illness presented to Kampala International University Teaching Hospital with a nine‐week history of migratory polyarthralgia, initially affecting the right knee and later involving the left knee, ankles, tarsal and metatarsal joints, and elbows. Pain worsened with passive movement and was associated with intermittent low‐grade fever, generalized weakness, pruritic rash, and tender cervical and axillary lymphadenopathy. The skin lesions appeared approximately two weeks after the onset of systemic symptoms and persisted throughout the illness without complete resolution. He denied morning stiffness, visual changes, oral ulcers, prior similar skin lesions, recent new medication use, or family history of autoimmune disease.

**FIGURE 1:**
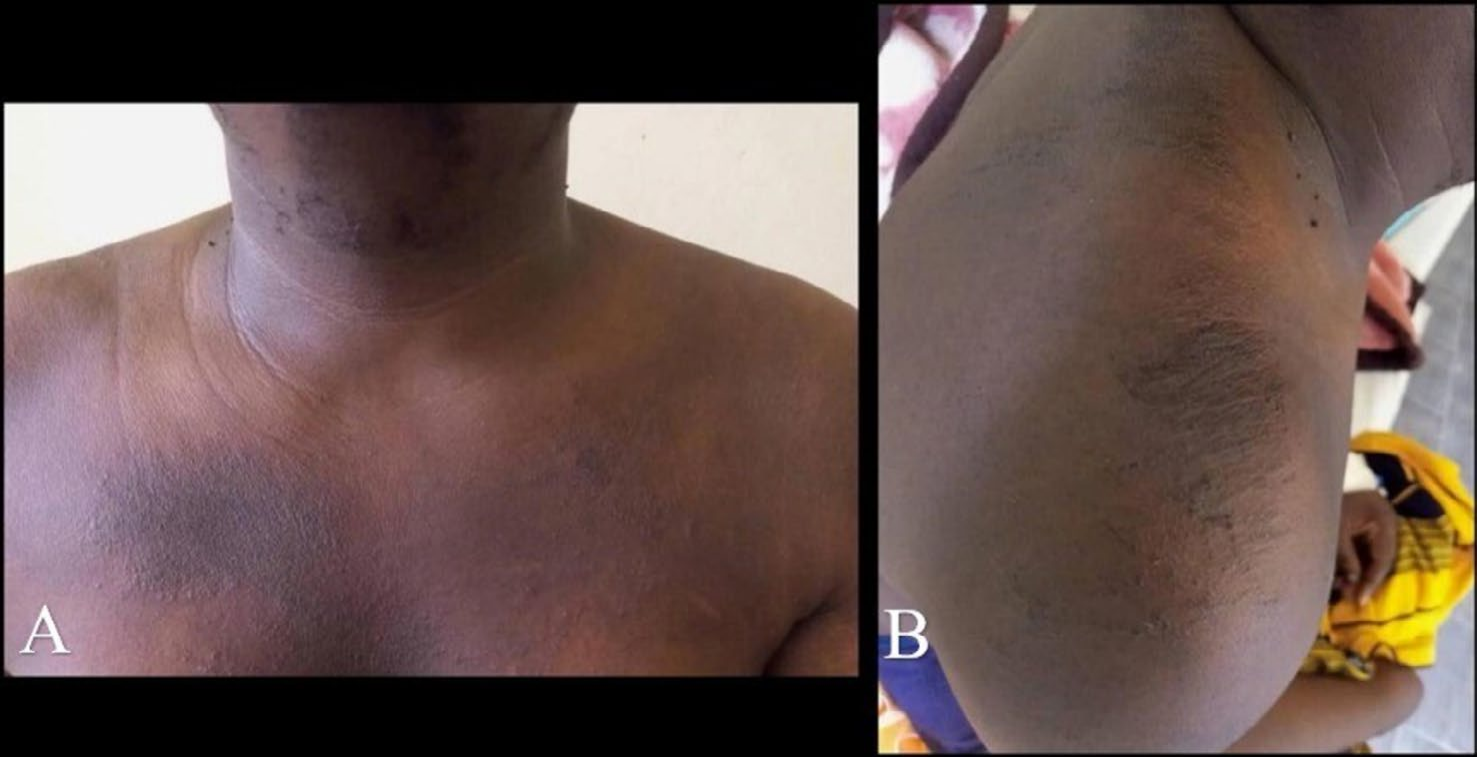
Persistent atypical cutaneous manifestations in adult‐onset Still's disease. (A) Well‐demarcated, persistent pruritic hyperpigmented plaque with mild crusting over the upper chest. (B) Persistent hyperpigmented, mildly crusted plaque involving the shoulder region. These lesions represent atypical, nonevanescent cutaneous manifestations associated with adult‐onset Still's disease.

On examination, temperature was 38.1°C. Small tender cervical and axillary lymph nodes were palpable. Dermatologic assessment by a dermatologist demonstrated well‐demarcated, persistent, crusted hyperpigmented plaques over the upper chest and shoulder, without mucosal involvement (Figure 1A,B). Musculoskeletal examination showed tenderness over affected joints without swelling, erythema, warmth, or deformity. Other systemic examinations, including cardiovascular, respiratory, and abdominal assessments, were unremarkable.

### 2.2 Investigations and Differential Diagnosis

Laboratory evaluation revealed leukocytosis (14.34 × 10^9^/L; reference range 4–11 × 10^9^/L) with neutrophil predominance, markedly elevated C‐reactive protein (CRP) (225.88 mg/L; reference < 10 mg/L), and hyperferritinemia (2000 ng/mL; reference range 20–300 ng/mL). Liver and renal function tests were within normal limits. Rheumatoid factor, anti‐cyclic citrullinated peptide antibodies, malaria testing, and Brucella serology were negative. Screening for HIV, hepatitis B and C, and syphilis was also negative. Knee radiographs were unremarkable. A skin biopsy was not performed due to limited local access to dermatopathology services; the diagnosis was based on dermatologic specialist assessment and characteristic clinical features.

Differential diagnoses considered included drug eruption, chronic dermatitis/eczema, urticarial vasculitis, erythema dyschromicum perstans, and cutaneous fungal infection. Drug eruption was unlikely given the absence of recent medication exposure. Urticarial vasculitis was considered less likely due to the absence of wheals, purpura, or residual vasculitic changes, while erythema dyschromicum perstans was unlikely given the acute systemic inflammatory presentation and markedly elevated inflammatory markers. Chronic dermatitis and fungal infection were inconsistent with acute systemic symptoms, laboratory findings and the morphology of the lesions. The presence of persistent hyperpigmented plaques in association with systemic inflammatory features was clinically compatible with an atypical cutaneous manifestation of AOSD.

Based on the Yamaguchi criteria: major features (fever, arthralgia, leukocytosis) and minor features (lymphadenopathy, atypical rash, negative serology) and after exclusion of infectious, autoimmune, and malignant causes, a diagnosis of moderate systemic AOSD was made [4].

### 2.3 Treatment and Outcome

The patient received intravenous diclofenac, intravenous omeprazole, and topical clobetasol, followed by an oral prednisone taper. Fever resolved within one week, joint pain improved substantially, pruritus subsided, the hyperpigmented plaques began to regress and lymphadenopathy resolved. He was discharged on oral diclofenac, omeprazole, and a prednisone taper with outpatient follow‐up scheduled. At three‐month follow‐up, the patient remained afebrile with no recurrence of joint symptoms, and the hyperpigmented plaques had significantly lightened without new lesions.

## 3 Discussion

### 3.1 Atypical Cutaneous Manifestations of Adult‐Onset Still's Disease

Adult‐onset Still's disease (AOSD) is classically associated with a transient, evanescent salmon‐pink rash that coincides with febrile episodes. However, persistent atypical cutaneous manifestations, including pruritic hyperpigmented or violaceous plaques, have been increasingly recognized as part of the disease spectrum [5, 6]. These lesions differ from the classic rash in that they may persist independently of fever and may resemble chronic inflammatory dermatoses, contributing to diagnostic delay.

Several case series have reported that non‐evanescent hyperpigmented plaques in AOSD tend to occur during periods of active systemic inflammation and may parallel disease activity, as reflected by elevated ferritin levels [5, 6]. In the present case, the temporal relationship between systemic symptoms and skin lesion onset, their persistence during periods of active disease, and their improvement with systemic therapy support interpretation of the lesions as a disease‐related inflammatory manifestation rather than a primary dermatologic disorder.

### 3.2 Diagnostic Considerations and Differential Diagnosis

The diagnosis of AOSD remains clinical and relies on the exclusion of infectious, autoimmune, and malignant conditions [4]. Typical laboratory findings include neutrophilic leukocytosis, markedly elevated acute‐phase reactants, and hyperferritinemia, reflecting intense systemic inflammation [7]. In this patient, fulfillment of the Yamaguchi criteria provided a robust diagnostic framework in the absence of disease‐specific biomarkers.

Persistent hyperpigmented plaques in a systemic inflammatory context raise important differential diagnoses. Urticarial vasculitis typically presents with recurrent wheals, purpura, or residual vasculitic changes, features absent in this case, making this diagnosis less likely [8]. Erythema dyschromicum perstans is characterized by slowly progressive gray‐brown macules without associated systemic inflammation, contrasting with the acute inflammatory presentation observed here [9]. The exclusion of these conditions, together with clinical response to systemic therapy, supports an AOSD‐related cutaneous process.

In resource‐limited settings, access to dermatopathology may be restricted. In such contexts, careful clinicopathologic correlation, multidisciplinary assessment, and therapeutic response remain essential components of diagnosis.

### 3.3 Pathophysiologic Insights Into Persistent Skin Involvement

AOSD is driven by dysregulated innate immune activation, characterized by excessive production of pro‐inflammatory cytokines, particularly interleukin (IL)‐1, IL‐6, and IL‐18 [10]. These cytokines promote neutrophil activation, endothelial dysfunction, and keratinocyte injury, contributing to both systemic and cutaneous inflammation [11]. Persistent cytokine signaling may result in interface dermatitis and post‐inflammatory hyperpigmentation, explaining the development of fixed or slowly resolving plaques rather than transient eruptions [12].

The improvement of cutaneous lesions following systemic corticosteroid therapy further supports a cytokine‐mediated inflammatory mechanism underlying persistent skin involvement in AOSD rather than an independent dermatologic condition.

### 3.4 Management and Clinical Implications

Early initiation of nonsteroidal anti‐inflammatory drugs and systemic corticosteroids remains the cornerstone of treatment for moderate systemic AOSD [13]. Prompt therapy is associated with rapid resolution of fever, improvement in joint symptoms, and gradual regression of cutaneous manifestations. In this patient, sustained clinical improvement at follow‐up suggests effective disease control.

This case underscores the importance of recognizing persistent atypical skin lesions as potential indicators of systemic inflammatory disease, particularly in resource‐constrained environments where diagnostic delays are common. In such settings, careful clinical assessment, systematic use of validated criteria such as the Yamaguchi criteria, and multidisciplinary collaboration can enable diagnosis even in the absence of advanced immunologic or histopathologic investigations. Awareness of these atypical presentations may facilitate earlier diagnosis and timely initiation of appropriate therapy.

## 4 Conclusion

Persistent atypical hyperpigmented skin lesions may represent an underrecognized cutaneous manifestation of adult‐onset Still's disease and can pose a diagnostic challenge by mimicking primary dermatologic conditions. Recognition of these atypical presentations, in conjunction with systemic inflammatory features and application of validated clinical criteria such as the Yamaguchi criteria, is essential for timely diagnosis and appropriate management, particularly in resource‐limited settings.

## Author Contributions

**Abdisalam Ahmed Sandeyl:** conceptualization, investigation, project administration, writing – original draft, writing – review and editing. **Stephen Kizito Mirembe:** resources, writing – review and editing. **Kusemererwa Byaruhanga:** data curation, investigation. **Farah Dubad Abdi:** investigation, writing – review and editing. **Abdisamad Guled Hersi:** investigation, writing – review and editing. **Louis K. Kamyuka:** investigation, resources. **Venance Emmanuel Mswelo:** conceptualization, writing – review and editing. **Rawan Yousif Hassan:** resources, supervision, writing – review and editing.

## Funding

The authors have nothing to report.

## Consent

Written informed consent was obtained from the patient for publication of this case report and any accompanying images.

## Conflicts of Interest

The authors declare no conflicts of interest.

## Data Availability

All data supporting the findings of this study are included within the manuscript. Additional information is available from the corresponding author upon reasonable request.
